# Automated and Enclosed Three-Dimensional Biofabrication System for Mesenchymal Stem Cell Culture to Enhance Diabetic Wound Healing

**DOI:** 10.34133/bmr.0205

**Published:** 2025-05-26

**Authors:** Yanmei Chen, Yang Xu, Jiawei Cai, Marianne Lauwers, Liwei Xiang, Yali Zheng, Hua Chu, Xianglong Chen, Dai Fei Elmer Ker, Cheng Zhang, Dan Michelle Wang, Zhiyong Zhang

**Affiliations:** ^1^ Translational Research Centre of Regenerative Medicine and 3D Printing, Department of Orthopaedic Surgery, Guangzhou Key Laboratory of Spine Disease Prevention and Treatment, Guangdong Province Engineering Research Center for Biomedical Engineering, State Key Laboratory of Respiratory Disease, Guangdong Provincial Key Laboratory of Major Obstetric Diseases, Guangdong Provincial Clinical Research Center for Obstetrics and Gynecology, The Third Affiliated Hospital, Guangzhou Medical University, Guangzhou, Guangdong, China.; ^2^ School of Biomedical Sciences, Faculty of Medicine, The Chinese University of Hong Kong, Hong Kong SAR, China.; ^3^ Institute for Tissue Engineering and Regenerative Medicine, The Chinese University of Hong Kong, Hong Kong SAR, China.; ^4^ Centre for Neuromusculoskeletal Restorative Medicine, Hong Kong Science Park, Hong Kong SAR, China.; ^5^ Department of Biomedical Engineering, Faculty of Engineering, The Hong Kong Polytechnic University, Hong Kong SAR, China.; ^6^ Department of Orthopaedics and Traumatology, The Chinese University of Hong Kong, Hong Kong SAR, China.

## Abstract

The industrialization of mesenchymal stem cells for regenerative medicine faces substantial challenges, particularly in large-scale production. Conventional 2-dimensional (2D) culture systems demonstrate limitations in meeting clinical requirements, such as inadequate cell yield, and poor cell–cell and cell–matrix interactions. These challenges can potentially be addressed by employing a 3D culture platform, which offers higher cell yields and enhanced efficacy. Moreover, it is essential to conduct a systematic and rigorous evaluation of cells produced in 3D culture systems to ensure their successful clinical translation. In this study, we cultured human umbilical cord mesenchymal stem cells (hUCMSCs) using an automated, scalable, and enclosed 3D microcarrier-bioreactor system, and comprehensively investigated their biological characteristics and potential therapeutic effects for diabetic wound repair. Our findings revealed that hUCMSCs harvested from this 3D microcarrier-bioreactor system are genetically stable and maintain the trilineage differentiation potential. Compared to hUCMSCs expanded under 2D conditions, those cultured in 3D exhibited reduced senescence and enhanced capabilities in migration, angiogenesis, and anti-inflammatory responses across different passages in vitro. RNA-sequencing analysis showed higher expression levels of genes related to angiogenesis and anti-inflammatory pathways in hUCMSCs cultured in 3D compared to those in 2D, which was further validated using quantitative real-time polymerase chain reaction and Western blot analysis. Additionally, 3D-cultured hUCMSCs demonstrated superior therapeutic effects for diabetic wound repair in mice, potentially due to their enhanced angiogenetic and anti-inflammatory effects. Collectively, our finding showcases the high quality of hUCMSCs cultured using an automated and enclosed 3D microcarrier-bioreactor system and their promising potential for diabetic wound repair.

## Introduction

Mesenchymal stem cells (MSCs) have garnered considerable attention in regenerative medicine, particularly for their potential in treating diabetic wounds [1]. These cells are characterized by low immunogenicity, minimal ethical concerns, and a reduced risk of tumorigenesis, which underlines their promise in this field [2]. The poor healing of diabetic skin wounds remains a significant clinical challenge, with research indicating that this is largely due to underlying pathological mechanisms such as decreased local growth factor secretion, oxidative stress, vascular damage, and chronic inflammatory responses [3]. Additionally, factors like impaired angiogenesis and persistent inflammation further complicate wound healing [4]. In this context, stem cell therapy holds great promise, given its ability to modulate inflammatory responses, secrete essential growth factors, and enhance vascularization, thereby promoting effective healing in diabetic wounds [5,6].

Among the diverse types of MSCs, umbilical cord mesenchymal stem cells (UCMSCs) stand out due to their distinct advantages over MSCs derived from other tissues [7]. UCMSCs are isolated from Wharton’s Jelly, a gelatinous substance surrounding umbilical vessels [7]. In contrast to the invasive procedures required for harvesting bone marrow and adipose tissue—which carry risks of pain and infectious complications—umbilical cord collection is noninvasive, is painless, and raises minimal ethical issues as it is derived from medical waste that are discarded at birth [8,9]. Furthermore, UCMSCs demonstrate superior proliferation and clonogenicity capacities relative to other MSCs [8,9]. This enhanced capacity can be attributed to their more primitive state compared with adult tissue-derived MSCs [10]. As of February 2025, there were 39 registered clinical trials investigating the application of UCMSCs in diabetes (30 trials) and wound healing (9 trials) (clinicaltrials.gov). Preliminary clinical data indicate that UCMSCs effectively accelerate diabetic wound regeneration while providing benefits such as reducing inflammation and improving angiogenesis in affected patients (NCT04104451; NCT03386708). Despite their benefits, the clinical translation of UCMSC therapies presents several challenges. Effective treatment often necessitates large quantities of cells—typically several million per kilogram of body weight—yet the yield from tissue sources is often limited [11]. Moreover, repeated passaging induces cellular senescence, thereby compromising the therapeutic properties of the cells [12].

The culture conditions of UCMSCs are a critical factor in MSC therapy, significantly influencing their proliferation, viability, and differentiation. Optimizing these conditions holds great promise for achieving large-scale expansion of UCMSCs while preserving their therapeutic properties [13]. Currently, most UCMSCs used in clinical trials are produced using conventional 2-dimensional (2D) culture systems [14]. Although the 2D culture production methodology meets the requirements for early-stage clinical trials, the cell yield is inadequate for commercial therapeutic applications, necessitating repeated passages leading to cell senescence [15]. The 2D culture systems also fail to accurately mimic the complex biophysical features of in vivo niche, such as reduced interactions with the extracellular matrix (ECM). Disruption of the ECM and stem cell interaction can reduce stem cell potency and compromise regenerative outcomes [15].

Advancements in 3D culture technologies offer a promising solution for enhancing the therapeutic properties of MSCs. A significant body of research has demonstrated that 3D culture can facilitate large-scale cell expansion of MSCs while positively influencing their therapeutic efficacy and potency [16]. Various 3D culture strategies exist, such as self-assembled cell spheroids and tissue-engineered microcarriers [17]. Among these, microcarrier systems present unique advantages, enabling the cultivation of adherent cell cultures in a suspension system that facilitates scalable expansion. Moreover, dissolvable and microporous microcarriers have shown up to 98% cell recovery while maintaining multipotency [18]. Their porous structure creates a microenvironment conducive to essential processes such as cell adhesion, proliferation, migration, and activation [19]. This innovative approach not only supports the growth of MSCs but also enhances their functional capabilities, making them more effective for therapeutic applications. As the cell therapy industry continues to develop vigorously, regulatory requirements from relevant agencies concerning production, quality stability, and technical standards for cell therapy products are becoming increasingly stringent. For example, strict quality control of materials used in cell manufacturing is one major requirement set forth in the Investigational New Drug (IND) application process for cell and gene therapy drugs in the United States. IND filings mandate the use of animal-free manufacturing materials whenever possible to reduce the risk of introducing exogenous viruses [20].

**Fig. 1.**
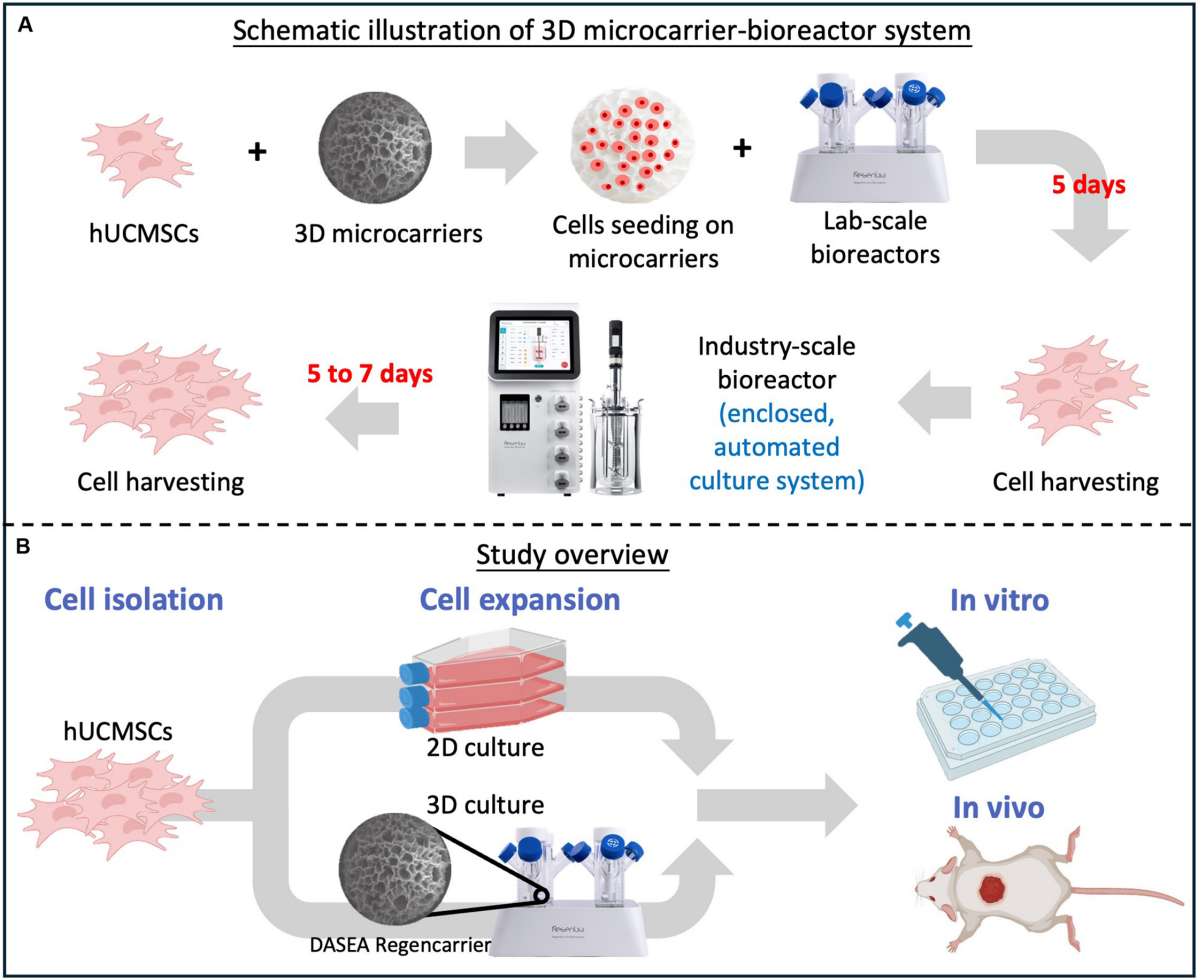
Study schematic and overview. (A) The diagram shows the culture process of the UCMSCs in the 3D microcarrier-bioreactor culture system. (B) The diagram shows the workflow for the in vitro and in vivo experiments, characterizing hUCMSCs cultured in both 2D and 3D microcarrier-bioreactor culture systems, performed in this study.

Our laboratory has previously developed a digital, automated, scalable, enclosed, and activated (DASEA) Regenbio bioreactor and microcarriers system (REGEN-αGEEK Medical Technology Co. Ltd., China) (Fig. 1A). The microcarriers are primarily composed of recombinant humanized collagen type I and range in size from 125 to 250 μm, featuring a porosity of ≥90% and pore diameters of ≥20 μm [China’s Centre for Drug Evaluation (CDE) registration number: F20230000567]. These microcarriers are pharmaceutical excipient-grade, non-animal origin [animal origin free (AOF)] recombinant humanized collagen type I microcarriers specifically designed for cell manufacturing and therapy [18]. They have received Master File (MF) qualification record by the Center for Biologics Evaluation and Research (CBER) in U.S. Food and Drug Administration (FDA), with filing numbers MF30042 and MF039036. These recombinant collagen I-based microcarriers exhibit excellent characteristics, including biocompatibility, biodegradability, and low immunogenicity, making them ideal for various applications in cell therapy [21]. The bioreactor system is equipped with 4 high-precision peristaltic pumps, multiple gas supply options (Air, O₂, N₂, CO₂), and precise fluid control mechanisms. This design allows uniform flow distribution, minimizes shear stress, and reduces cell damage. Additionally, it offers flexible operational modes for monitoring and controlling the culture process, which has been proved to improve production efficiency while ensuring consistency in cell culture [22]. For the successful clinical translation of MSCs manufactured using this 3D microcarrier-bioreactor system, it is essential to conduct a systematic and rigorous evaluation of the generated MSCs. In this study, we evaluated human umbilical cord mesenchymal stem cells (hUCMSCs) cultured within this system using the following key assessment criteria: MSC quality (preservation of stem cell characteristics), biosafety (evaluation of the risk of malignant cell formation), and the bio-efficacy of stem cells (assessment of the clinical therapeutic efficacy) for diabetic wound healing [5] (Fig. 1B). Our results demonstrate that hUCMSCs cultured in this 3D microcarrier-bioreactor system exhibit genetic stability and trilineage differentiation potential. Moreover, compared with hUCMSCs cultured in 2D conditions, 3D-cultured hUCMSCs exhibited reduced senescence and enhanced capabilities in migration, angiogenesis, and anti-inflammatory responses across different passages (P4 and P9) in vitro. RNA-sequencing (RNA-seq) analyses revealed distinct gene expression profiles between hUCMSCs cultured in 2D and 3D, with 3D-cultured cells showing higher expression levels of genes related to angiogenesis and anti-inflammatory pathways, which were validated with quantitative real-time polymerase chain reaction (qRT-PCR) and Western blot (WB) analysis in vitro. Additionally, in vivo studies demonstrated that 3D-cultured hUCMSCs accelerated wound healing in diabetic mice, significantly enhancing angiogenesis and anti-inflammatory capabilities compared to 2D-expanded hUCMSCs. The combination of advanced automation, precise environmental control, and innovative microcarrier design makes this 3D microcarrier-bioreactor system particularly well-suited for industrial-scale MSC production, improving both the quality and therapeutic effectiveness of MSC-based treatments.

## Materials and Methods

### Establishment of primary culture of hUCMSCs

Human umbilical cord tissue was obtained from a healthy donor in accordance with approved guidelines from the Institutional Ethics Review Board of Third Affiliated Hospital of Guangzhou Medical University (permit number: 2020_001). hUCMSCs were isolated using the tissue block adhesion method, as previously described [23]. In short, after washing and removing blood vessels and connective tissue, the umbilical cord was cut into small pieces, ranging approximately 1 to 8 cm^3^. These tissue blocks were cultured in serum-free UltraMedia (RGM0051, REGEN-αGEEK Biotechnology Co. Ltd., China) until scattered cells appear around the tissue block. These cells were considered P0 and were either stored at −80 °C or harvested for further expansion in a 2D or 3D microcarrier-bioreactor system.

### 2D expansion of hUCMSCs

In brief, 1.4 × 10^6^ primary hUCMSCs were inoculated into a T175 culture flask and cultured with serum-free UltraMedia (RGM0051, REGEN-αGEEK Biotechnology Co. Ltd., China). The medium was changed every other day. When the confluence reached 90%, the hUCMSC were digested with UltraTryple (RGM0061, REGEN-αGEEK Biotechnology Co. Ltd., Haining, China) and harvested for passaging and experiments.

### 3D expansion of hUCMSCs

Initially, around 2.0 × 10^6^ P1/P2 hUCMSCs were harvested from 2D culture and mixed with 100 mg of microcarriers (REGEN-αGEEK Biotechnology Co. Ltd., Haining, China). The mixture was then cultured in a 3D environment using a laboratory-scale mini DASEA bioreactor with a capacity of 125 ml (REGEN-αGEEK Biotechnology Co. Ltd., Haining, China). After 5 d, a sufficient cell density was achieved (6 × 10^7^ cells based on sampling inspection assessment) and then the cells were transferred to a larger DASEA bioreactor with a capacity of 2 l (REGEN-αGEEK Biotechnology Co. Ltd., Haining, China) to obtain greater cell quantities. Following the manufacturer’s instructions, the 3D culture conditions were programmed as follows: a temperature of 37 °C, a pH of 7.3, and an oxygen level of 40%. The initial agitation was set at 50 rpm for 5 min, followed by a 30-min period with no agitation. From day 1 to day 7, the agitation speed was maintained at 60 rpm. The serum-free UltraMedia (RGM0051, REGEN-αGEEK Biotechnology Co. Ltd., China) was replenished to 2 l after the initial 24 h, and thereafter, 50% of the medium was replaced with fresh medium every 48 h before harvesting cells on day 7 (Fig. 1A). Cells were harvested and characterized at P4 (to represent early passage cells) and P9 (to represent late passage cells) [24,25]. hUCMSCs at passages 4 and 9 were used for quality assurance (QA) characterizations, while hUCMSCs at P4 were used in all experiments to validate therapeutic efficacy.

Prior to harvesting, the cell–microcarrier complexes were allowed to settle by ceasing the agitation. The medium was removed, and 500 ml of phosphate-buffered saline (PBS) was added to the bioreactor, maintaining a temperature of 37 °C. A lysis reagent (MC001LS, REGEN-αGEEK, China) was prepared at an enzyme-to-microcarrier ratio of 0.2 mg of enzyme per 1 mg of microcarriers. Lysis was conducted at 37 °C for 30 to 40 min, starting with an agitation speed of 50 rpm, which was gradually reduced by 10 rpm every 10 min, reaching a minimum of 30 rpm. The resulting cell suspension was collected and centrifuged at 300*g* for 5 min. After discarding the supernatant, the cells were resuspended in PBS and washed by centrifugation at 300*g* for another 5 min. This washing step was repeated twice more with PBS, after which the cells were counted and prepared for subsequent experiments.

### Flow cytometry analysis

Using flow cytometry, the 3D-cultured hUCMSCs were characterized (tune NxT, Thermo Fisher). Positive markers, i.e., CD73-FITC (fluorescein Isothiocyanate) (BD Pharmingen, 1:20), CD90-FITC (BD Pharmingen, 1:50), and CD105-PE (phycoerythrin) (BD Pharmingen, 1:20), and negative markers, i.e., CD11b-FITC (BD Pharmingen, 1:20), CD19-FITC (BD Pharmingen, 1:5), CD45-FITC (BD Pharmingen, 1:5), CD34-PE (BD Pharmingen, 1:5), and HLA-DR-PerCy5 (BD Pharmingen, 1: 5), were determined. Data were analyzed using FlowJo V7.6.2 software (BD Biosciences).

### Trilineage differentiation assay

The hUCMSCs cultured using the 3D microcarrier-bioreactor system were evaluated for their trilineage differentiation. The cells were differentiated to the adipogenic (HUXUC-90031), osteogenic (HUXUC-90021), or chondrogenic (HUXUC-90041) (OriCell, Guangzhou, China) lineage following the manufacturer’s instructions. After induction, cells were stained with Oil Red O staining (ChemCruz), Alizarin Red S staining (Electron Microscopy Sciences, Hatfield, PA, USA), and Alcian Blue staining (Electron Microscopy Sciences) for the detection of cell differentiation as previously described [26].

### Soft agar colony formation assay

The soft agar colony formation assay was performed to evaluate malignant cellular transformation. Therefore, 0.7% and 1% agarose (Biofroxx, Germany) in ultrapure water were prepared and autoclaved. The 1% agarose solution was mixed with an equal volume of 2× Dulbecco’s modified Eagle’s medium (DMEM) (VivaCell BIOSCIENCES, Shanghai, China) containing 20% fetal bovine serum (FBS), quickly added to a 6-well plate (4 ml per well), and solidified at room temperature as the base layer. The 0.7% agarose solution was combined with an equal volume of 2× DMEM containing 20% FBS to obtain a 0.35% agarose mixture. Five thousand cells (harvested from 3D culture) were mixed with the 0.35% agarose mixture and uniformly seeded onto the solidified base layer at a volume of 2 ml per well. HeLa cells were used as positive control. After solidifying at room temperature, the plates were incubated at 37 °C in a 5% CO_2_ incubator for 16 d and observed under a microscope.

### Cell chromosome analysis

The 3D-cultured hUCMSCs were cryopreserved for a standard chromosome analysis at a resolution of 300 bands, following the manufacturer’s instructions as previously described [27]. In brief, the chromosomes are captured with a light microscope. Then, the image of each chromosome was segmented and extracted from the microscopic image of metaphase chromosome. Finally, the extracted chromosomes are classified and sorted to form a karyotype image with 24 types of chromosomes.

### Short tandem repeat analysis

The 3D-cultured hUCMSCs were harvested, and a short tandem repeat (STR) analysis was performed. Genomic DNA from hUCMSCs was isolated using a genome extraction kit (Axygen, USA) and amplified by the STR amplification protocol. The STR loci and sex gene Amelogenin were detected by an ABI 3730XL Genetic Analyzer. The STR data were compared with the ExPASy database, as previously described [28].

### Telomerase activity

RNA (1 μg) was extracted from the 3D-cultured hUCMSCs using TRIzol Reagent (Invitrogen, Thermo Fisher, K1622, USA), and the telomerase was reconstituted by expressing human telomerase reverse transcriptase (TERT) protein and telomerase RNA by using the TnT transcription/translation system (Promega, Madison, WI), and its activity was quantified by the Cy5-fluorescent gel-based telomeric repeat amplification protocol (TRAP) assay with the TERT Real-Time Quantitative Assay Kit (BPT50, Biowing, China), following the manufacturer’s instructions [29].

### Senescence assay

hUCMSCs from both the 2D culture and the 3D microcarrier-bioreactor system were harvested and seeded onto 6-well plates at a density of 5 × 10^4^ cells/well and cultured until reaching 90% confluency. The cells were then fixed with 4% (v/v) paraformaldehyde for 10 min and stained with senescence-associated β-galactosidase (SA-β-gal) solution (C0602, Beyotime, Shanghai, China) for 12 h. The SA-β-gal-positive cells, stained in blue, were observed under a microscope, and the stained areas were quantified with ImageJ software.

### Transwell assay

hUCMSCs from both the 2D culture and the 3D microcarrier-bioreactor system were harvested and suspended in DMEM/F12 serum-free medium (Gibco, USA) with a density of 1.5 × 10^5^ cells/ml. The cell suspension (200 μl) was added to each upper chamber of a Transwell 24-well plate (8 μm pore size, Corning, USA), and 800 μl of DMEM/F12 medium with 10% FBS was added to the lower chamber. After a 24-h incubation at 37 °C with 5% CO_2_, the cells attached to the lower surface of the Transwell chamber were fixed with 4% paraformaldehyde. After 30 min, the cells were washed with PBS and stained for 10 min with 2.5% crystal violet solution (G1061, Solarbio, China).

### Tube formation assay

Conditioned media (CM) were prepared by collecting the supernatants of hUCMSCs cultured from both the 2D culture and the 3D microcarrier-bioreactor system, followed by centrifugation at 2,000*g* for 20 min at 4 °C to remove any remaining cells and cell debris. Subsequently, an ultrafiltration device (Millipore, USA, 100 kDa molecular weight cutoff) was used to concentrate the condition medium. Human umbilical vein endothelial cells (HUVECs; Procell, China) were seeded at a density of 5 × 10^3^ cells per 96-well onto a precoated Matrigel matrix (50 μl/well, Corning USA) and cultured in the collected CMs. After 8 h of culture, images were captured under a microscope to examine the formation of tubular network structures. The ImageJ software was used to measure and quantify the number of branch points and the length of the main segments, as previously described.

### Coculture of hUCMSCs with M1 macrophages

RAW264.7 macrophages (Procell, China) were seeded onto 6-well plates at a density of 1 × 10^5^/cm^2^. After 12 h, lipopolysaccharide (LPS) (100 ng/ml) was added to each well and incubated overnight to induce polarization into M1 macrophages. Three experimental groups were established: M1 macrophages alone, M1 macrophages cocultured with hUCMSCs from 2D culture, and M1 macrophages together with hUCMSCs of the 3D microcarrier-bioreactor system. For the coculture, hUCMSCs were seeded at a concentration of 1 × 10^5^/cm^2^ in the upper chamber of Transwell, while the M1 macrophages were placed in the lower Transwell chambers at the same concentration. After 2 d of culture, macrophages were collected to assess the gene expression levels of M1 polarization-related genes [CD86, tumor necrosis factor-α (TNF-α), and interleukin-1β (IL-1β)] and M2 polarization-related genes [arginase-1 (ARG-1) and CD206] using qRT-PCR. Additionally, flow cytometry was performed to analyze macrophage phenotypes using markers including FITC-conjugated anti-mouse CD206 antibody (BioLegend, 141703, USA, 1:150) and PE-conjugated anti-mouse CD86 antibody (BioLegend, 159204, USA, 1:150), as previously described.

### qRT-PCR analysis

The total RNA of hUCMSCs from both the 2D culture and the 3D microcarrier-bioreactor system as well as the total RNA of the tissue samples from 2D-hUCMSC-treated and 3D-hUCMSC-treated wound skin was extracted according to the instructions of the Ultrapure RNA Kit (CWBIO, Jiangsu, China). qRT-PCR was performed using the SYBR Green qPCR kit [AG11718, Accurate Biotechnology (Hunan) Co. Ltd., Changsha, China] and the QuantStudio 3 Real-Time PCR System (Thermo Fisher, USA). Gene expression levels of angiogenic markers [epidermal growth factor (EGF), placental growth factor (PGF), early growth response 1 (EGR1), angiopoietin 1/2 (ANG1/2), vascular endothelial growth factor (VEGF), platelet-derived growth factor (PDGF), and hypoxia-inducible factor-1 (HIF-1α)], fibroblast activation markers [fibroblast growth factor 2 (FGF2) and type I collagen (collagen I)], inflammatory markers [interferon-inducible protein 1 (IFIT1), Kruppel-like factor 2 (KLF2), nuclear factor κB inhibitor Z (NFKBIZ), dual specificity phosphatase 1 (DUSP1), CD86, TNF-α, IL-1β, IL-4, IL-6, IL-10, CD163, CD206, and transforming growth factor-β (TGF-β1)], pluripotency genes [SRY-box transcription factor 2 (SOX2), octamer-binding transcription factor 4 (OCT4), and Nanog homeobox (Nanog)], and senescence-related genes [P53, P21, P19, and poly (ADP-ribose) polymerase 1 (PARP1)] were determined with glyceraldehyde-3-phosphate dehydrogenase (GAPDH) as reference gene. The relative gene expression levels were quantified using the 2^−ΔΔCt^ method. The primer sequences used in this study are provided in Table 1.

**Table 1.** The primer sequences for qRT-PCR

	Forward 5′-3′	Reverse 5′-3′
Mouse CD86	TCAATGGGACTGCATATCTGCC	GCCAAAATACTACCAGCTCACT
Mouse TNF-α	CAGGCGGTGCCTATGTCTC	CGATCACCCCGAAGTTCAGTAG
Mouse IL-1β	GAAATGCCACCTTTTGACAGTG	TGGATGCTCTCATCAGGACAG
Mouse IL-10	CTTACTGACTGGCATGAGGATCA	GCAGCTCTAGGAGCATGTGG
Mouse CD163	GGTGGACACAGAATGGTTCTTC	CCAGGAGCGTTAGTGACAGC
Mouse CD206	CTCTGTTCAGCTATTGGACGC	TGGCACTCCCAAACATAATTTGA
Mouse ARG-1	CTCCAAGCCAAAGTCCTTAGAG	GGAGCTGTCATTAGGGACATCA
Mouse FGF2	AGGGCTCTGGTGGATGAATTA	CCATCTGGATTGATTCGGAAGGA
Mouse HIF-1α	TCTCGGCGAAGCAAAGAGTC	AGCCATCTAGGGCTTTCAGATAA
Mouse Collagen I	GCTCCTCTTAGGGGCCACT	ATTGGGGACCCTTAGGCCAT
Mouse IL-6	CTGCAAGAGACTTCCATCCAG	AGTGGTATAGACAGGTCTGTTGG
Mouse TGF-β1	CCACCTGCAAGACCATCGAC	CTGGCGAGCCTTAGTTTGGAC
Mouse Angiopoietin1(ANG1)	ATCCCGACTTGAAATACAACTGC	CTGGATGATGAATGTCTGACGAG
Mouse VEGF	CTGCCGTCCGATTGAGACC	CCCCTCCTTGTACCACTGTC
Mouse PDGF	CATCCGCTCCTTTGATGATCTT	GTGCTCGGGTCATGTTCAAGT
Mouse PGF	AGTGGAAGTGGTGCCTTTCAA	GTGAGACACCTCATCAGGGTA
Mouse EGF	AGAGCATCTCTCGGATTGACC	CCCGTTAAGGAAAACTCTTAGCA
Mouse GAPDH	AGGTCGGTGTGAACGGATTTG	GGGGTCGTTGATGGCAACA
Human GAPDH	GCATCTTCTTTTGCGTCG	TGTAAACCATGTAGTTGAGGT
Human SOX2	TGGCGAACCATCTCTGTGGT	CCAACGGTGTCAACCTGCAT
Human OCT4	TCGAGAAGGATGTGGTCCGA	GCCTCAAAATCCTCTCGTTG
Human Nanog	CCTGTGATTTGTGGGCCTG	GACAGTCTCCGTGTGAGGCAT
Human P53	CAGCACATGACGGAGGTTGT	TCATCCAAATACTCCACACGC
Human P21	CGATGGAACTTCGACTTTGTCA	GCACAAGGGTACAAGACAGTG
Human P19	GATCCAGGTGGGTAGAAGGTC	CCCCTGCAAACTTCGTCCT
Human PARP1	CGGAGTCTTCGGATAAGCTCT	TTTCCATCAAACATGGGCGAC
Human IL- 10	GACTTTAAGGGTTACCTGGGTTG	TCACATGCGCCTTGATGTCTG
Human IL-4	CCAACTGCTTCCCCCTCTG	TCTGTTACGGTCAACTCGGTG
Human TGF-β1	GGCCAGATCCTGTCCAAGC	GTGGGTTTCCACCATTAGCAC
Human HIF-1α	GAACGTCGAAAAGAAAAGTCTCG	CCTTATCAAGATGCGAACTCACA
Human VEGF	AGGGCAGAATCATCACGAAGT	AGGGTCTCGATTGGATGGCA
Human PDGF	CTCGATCCGCTCCTTTGATGA	CGTTGGTGCGGTCTATGAG
Human FGF2	AGAAGAGCGACCCTCACATCA	CGGTTAGCACACACTCCTTTG
Human EGF	TGGATGTGCTTGATAAGCGG	ACCATGTCCTTTCCAGTGTGT
Human PGF	GAACGGCTCGTCAGAGGTG	ACAGTGCAGATTCTCATCGCC
Human EGR1	GGTCAGTGGCCTAGTGAGC	GTGCCGCTGAGTAAATGGGA
Human Angiopoietin1 (ANG1)	AACTTTCGGAAGAGCATGGAC	CGAGTCATCGTATTCGAGCGG
Human Angiopoietin2 (ANG2)	AACTTTCGGAAGAGCATGGAC	CGAGTCATCGTATTCGAGCGG
Human IL-6	ACTCACCTCTTCAGAACGAATTG	CCATCTTTGGAAGGTTCAGGTTG
Human IFIT1	TTGATGACGATGAAATGCCTGA	CAGGTCACCAGACTCCTCAC
Human KLF2	TTCGGTCTCTTCGACGACG	TGCGAACTCTTGGTGTAGGTC
Human NFKBIZ	AGAGGCCCCTTTCAAGGTGT	TCCATCAGACAACGAATCGGG
Human DUSP1	AGTACCCCACTCTACGATCAGG	GAAGCGTGATACGCACTGC

### RNA-seq

Total RNA was extracted from hUCMSCs cultured in 2D and 3D conditions at P4, and the concentration and quality of the RNA were evaluated. The RNA integrity number (RIN > 7) was determined using the Agilent Bioanalyzer 4150 (Agilent Technologies, CA, USA). mRNA was purified from 1 μg of total RNA, and differential expression analysis of mRNA between groups was performed using the DESeq2 package (http://bioconductor.org/packages/release/bioc/html/DESeq2.html). The default thresholds for differentially expressed genes were set as |log_2_FC| > 1 and *P* < 0.05, which served as the criteria for selecting candidate genes for subsequent analyses. These stringent selection criteria were employed to maximize the sensitivity of the analysis and facilitate a comprehensive screening of potential candidate genes. Gene Ontology (GO) and Kyoto Encyclopedia of Genes and Genomes (KEGG) enrichment analyses were conducted on the differentially expressed genes to elucidate their functional enrichment, providing insights into the functional differences between the samples at the gene level. The clusterProfiler R package was used to perform the GO and KEGG enrichment analyses, and *P* < 0.05 was considered to indicate significant enrichment of a particular GO term or KEGG pathway.

### WB analysis

To validate the protein production of hUCMSCs cultured in 2D and 3D settings, WB analysis was performed as previously described [30]. Briefly, protein samples (20 μg) were loaded onto the gel and separated by electrophoresis (90 V for stacking gel and 120 V for resolving gel). The gel was then soaked in transfer buffer (Sigma-Aldrich) and 1 g/l sodium dodecyl sulfate (Sigma-Aldrich) in dH_2_O. The protein was transferred onto a polyvinylidene difluoride membrane (Thermo Fisher Scientific) using a wet transfer system (Bio-Rad) and incubated with a primary antibody (IL-6 rabbit polyclonal antibody, 1:2,000, Proteintech, China, #21865-1-AP; VEGF rabbit polyclonal antibody, 1:1,000, Proteintech, China, #19003-1-AP; TGF-β1 rabbit recombinant antibody, 1:1,000, Proteintech, China, #81746-2-RR; GAPDH mouse monoclonal antibody, 1:1,000, Beyotime, China, #AF0006) and a secondary antibody (goat anti-rabbit secondary antibody, 1:5,000, Bio-Rad; goat anti-mouse secondary antibody, 1:5,000, Bio-Rad). The membrane was allowed to react with a chemiluminescence substrate (Thermo Fisher Scientific) and imaged using a ChemiDoc imaging system (Bio-Rad).

### Diabetic mouse wound healing model

Male C57BL/6J mice were obtained from the Guangdong Medical Laboratory Animal Centre (B202403-6). The mice were single injected with streptozotocin (STZ; 125 mg/kg, Sigma-Aldrich, St. Louis, MO, USA) in citrate buffer (pH 4.5) on day 1 to establish a diabetic model, as previously described [18]. Mice that exhibited persistent fasting blood glucose levels exceeding 16 mM 7 d after STZ treatment were considered to have successfully established diabetes. One week after STZ treatment, all diabetic mice underwent skin defect surgery and subcutaneous injections of 2D-hUCMSCs, 3D-hUCMSCs, or PBS around the defect area. The mice were anesthetized by intraperitoneal injection of 1% sodium pentobarbital at a dose of 50 mg/kg. Subsequently, a full-thickness skin excision wound with a diameter of 10 mm was created on the dorsal side of the torso using surgical scissors. One week after STZ treatment, 45 diabetic mice with a skin defect were randomly divided into 3 groups (*n* = 15 per group): (a) diabetic wound model control group: 0.1 ml of PBS was injected around the wound; (b) 2D-hUCMSC treatment group: 0.1 ml of PBS containing 1 × 10^6^ 2D-hUCMSCs was injected around the wound; (c) 3D-hUCMSC treatment group: 0.1 ml of PBS containing 1 × 10^6^ 3D-hUCMSCs was injected around the wound. The wound healing rate of the mice was assessed by photographing the wounds on 0, 3, 6, and 9 d post-surgery. The wound healing rate on day *n* was calculated as (initial wound area − wound area on day *n*)/initial wound area × 100%, using the ImageJ software.

### Histological analysis

All mice were sacrificed, and skin tissue samples were harvested on day 9. The tissue samples were fixed in 4% paraformaldehyde for 24 h, followed by paraffin embedding for 48 h. Slides with 5-μm thickness from paraffin-embedded skin tissues were stained by hematoxylin and eosin (H&E) and Masson’s trichrome staining and imaged by a light microscope.

### Immunohistochemical and immunofluorescence staining

On day 9 post-surgery, skin tissue samples were harvested for immunohistochemical (IHC) and immunofluorescence (IF) staining. The paraffin-embedded tissue sections were deparaffinized, and antigen retrieval was performed by heating the sections in a citrate buffer (pH 6.0). After 3 PBS washes, the samples were blocked with 5% bovine serum albumin (BSA) for 30 min, followed by overnight incubation with the respective primary antibodies (Table 2) at 4 °C. The sections were then washed 3 times with PBS and incubated with the corresponding secondary antibodies for 60 min in the dark. Bright and fluorescent images were captured using an inverted fluorescence microscope. The positively stained area in each field was quantified using the ImageJ software.

**Table 2.** Detailed information of primary antibodies for IHC and IF staining

Antibodies	Article no.	Company	Dilution
CD31 (rabbit)	GB113151	Servicebio (China)	1:300
α-SMA (rabbit)	GB111364	Servicebio	1:300
DAPI	H21491	Invitrogen	1:1,000
F4-80 (rabbit)	GB113373	Servicebio	1:500
CD206 (rabbit)	GB113497	Servicebio	1:200

### Statistical analysis

The GraphPad Prism 7.0 software was used for data processing and graph generation. All data are presented as mean ± standard error of the mean (SEM). One-way analysis of variance (ANOVA) was performed for comparisons among multiple groups, followed by Tukey’s post hoc test. For comparisons between 2 groups, Student’s *t* test was applied. Statistical significance was set at *P* < 0.05 for all tests.

## Results

### 3D culture in the microcarrier-bioreactor system supported the expansion of high-quality hUCMSCs

**Fig. 2.**
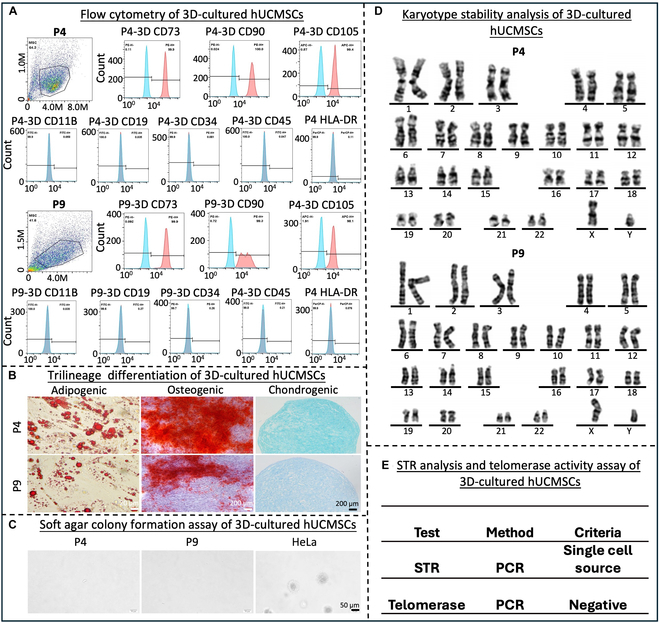
Characterization of 3D-cultured hUCMSCs. (A) Surface antigen profiling in 3D-cultured hUCMSCs by flow cytometry with positive markers CD73, CD90, and CD105, and negative markers CD11B, CD19, CD34, CD45, and HLA-DR. (B) Trilineage differentiation of hUCMSCs differentiated into adipocytes, osteocytes, and chondrocytes. Scale bar, 200 μm. (C) Soft agar colony formation assay on 3D-cultured hUCMSCs indicating lack of tumorigenicity compared to the HeLa cell line. Scale bar, 50 μm. (D) Chromosome analysis showed stable karyotype of 3D-cultured hUCMSCs. (E) STR analysis and telomerase activity assay convinced the nontumorigenicity and stability of 3D-cultured hUCMSCs. *n* = 3 independent assays, with each experiment comprising 3 technical replicates for every condition.

hUCMSCs (P4 and P9) cultured in the 3D microcarrier-bioreactor system were harvested and evaluated based on the criteria set by the International Society for Cell & Gene Therapy (ISCT). The presence of CD73, CD90, and CD105 and the absence of CD11b, CD19, CD45, CD34, and HLA-DR markers were confirmed in the hUCMSCs, at both P4 and P9 (Fig. 2A). The hUCMSCs also maintained the capacity to differentiate into adipogenic, osteogenic, and chondrogenic lineages demonstrated by positive Oil Red “O”, Alizarin Red S, and Alcian Blue staining as well as morphological characterization (Fig. 2B). Additionally, hUCMSCs underwent biosafety testing, including soft agar formation, karyotype stability analysis, STR profiling, and telomerase activity assays (Fig. 2C to E). G-banding karyotype analysis confirmed the genomic stability of 3D-expanded cells (Fig. 2D), while soft agar formation assays and telomerase activity assays indicated a lack of tumorigenicity (Fig. 2C). STR analysis further demonstrated that the hUCMSCs were not contaminated by other cell lines (Fig. 2E). These results suggest that hUCMSCs expanded through continuous 3D serial cultures meet the necessary quality requirements for stem cell therapy.

### 3D culture reduced senescence and maintained pluripotency of hUCMSCs

**Fig. 3.**
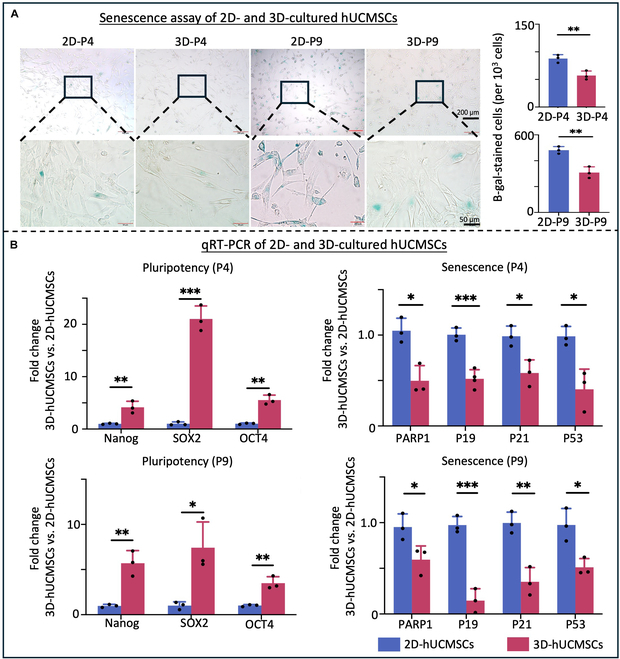
Assessment of senescence and stemness of 2D- and 3D-cultured hUCMSCs in vitro. (A) Senescence assay with β-galactosidase staining showed reduced number of senescent cells (stained blue) in 3D-cultured hUCMSCs at passage 4 (P4) and 9 (P9) compared to 2D-hUCMSCs. Scale bar, 50 μm. (B) qRT-PCR analysis demonstrated higher gene expression level of cell pluripotency-related genes (SOX2, OCT4, and Nanog) and lower expression of senescence-related genes (P53, P21, P19, and PARP1) in 3D-hUCMSCs compared with 2D-hUCMSCs, with GAPDH as the reference gene (mean ± SD; *n* = 3 independent assays, with each experiment comprising 3 technical replicates for every condition). *P < 0.05, **P < 0.01, ***P < 0.001.

Since long-term cell culture can lead to cellular senescence, SA-β-gal staining and qRT-PCR for senescent genes was performed to compare senescence of hUCMSCs cultured from the 2D and 3D microcarrier-bioreactor system. The SA-β-gal staining demonstrated significantly reduced numbers of senescent cells of 3D-cultured cells compared to 2D-cultured hUCMSCs (Fig. 3A). The gene expression level of P53, known for its critical role in cellular senescence [31], showed results consistent with the SA-β-gal staining. Notably, gene expression levels of senescence-associated secretory phenotype (SASP) markers (PARP1, P19, P21, and P53) were significantly lower in 3D-hUCMSCs compared to 2D-hUCMSCs (Fig. 3B). Concurrently, the higher gene expression level of pluripotency-related transcription factors [OCT4, SOX2, and NANOG (*P* < 0.05)] further proved that the 3D culture maintained the pluripotency and viability of hUCMSCs (Fig. 3B). These results indicate that the 3D microcarrier-bioreactor can reduce the senescence of hUCMSCs at higher passages and maintain their pluripotency.

### 3D culture promoted the angiogenesis and anti-inflammatory ability of hUCMSCs

**Fig. 4.**
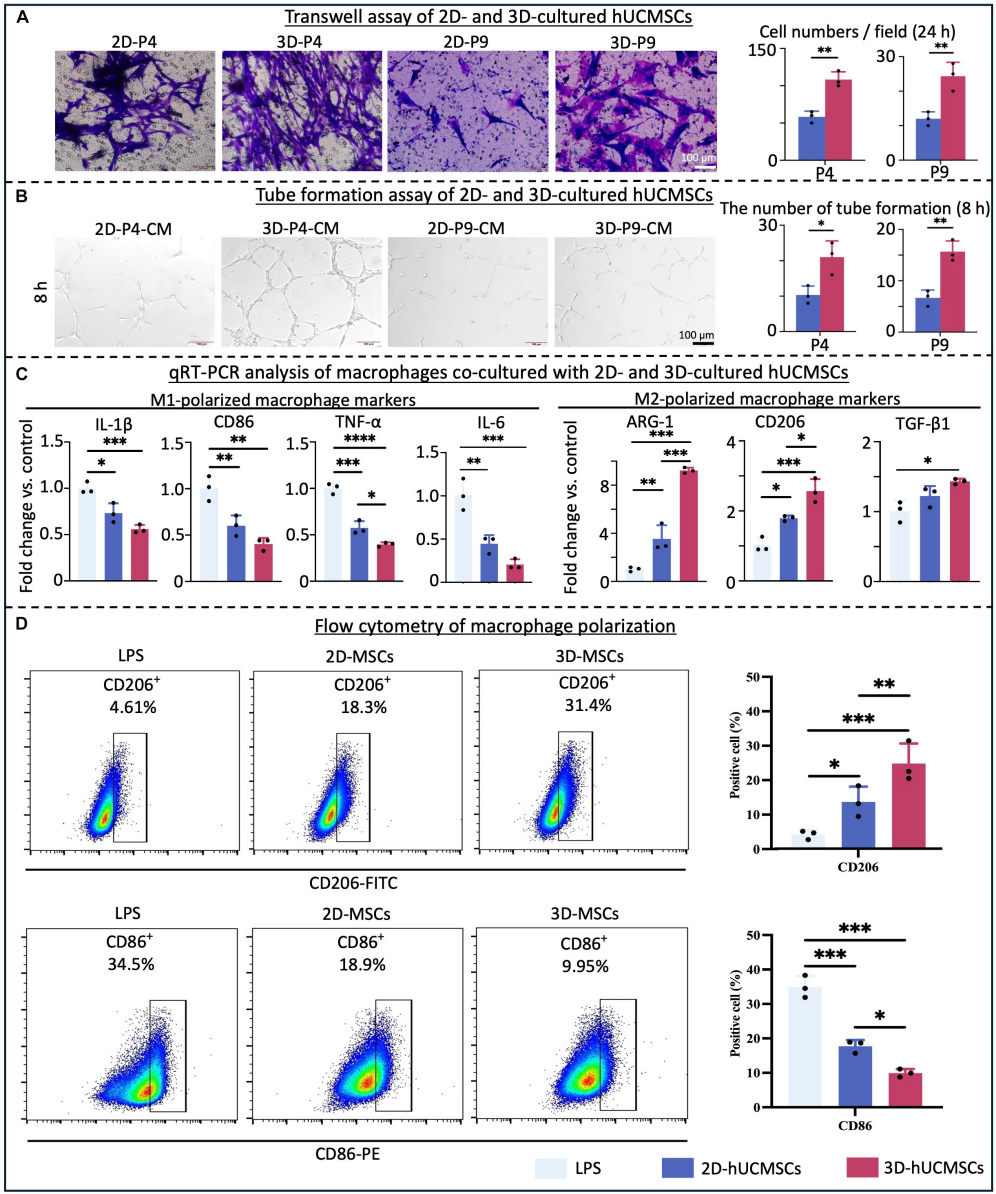
The assessment of migration, angiogenesis, and immunomodulation capacity of 2D- and 3D-cultured hUCMSCs in vitro. (A) Transwell assays showed higher cell migration ability of 3D-cultured hUCMSCs compared with 2D-cultured hUCMSCs. Scale bar, 100 μm. (B) Tube formation assay showed increased tube formation numbers in HUVECs from the 3D-CM group compared to the 2D-CM group. Scale bar, 100 μm. (C) qRT-PCR analysis demonstrated lower expression level genes related to M1 polarization (CD86, TNF-α, and IL-1β) and higher expression level of genes related to M2 polarization (ARG-1 and CD206) in macrophages cocultured with 3D-hUCMSCs compared with macrophages cocultured with 2D-hUCMSCs, with GAPDH as the reference gene. (D) Representative flow charts of flow cytometry for CD86 and CD206 on F4/80^+^ cells showed effects of coculture with 2D- and 3D-cultured hUCMSCs on macrophage polarization (mean ± SD; *n* = 3 independent assays, with each experiment comprising 3 technical replicates for every condition). *P < 0.05, **P < 0.01, ***P < 0.001, ****P < 0.0001.

Numerous studies have shown that MSCs can promote angiogenesis and modulate immune responses [18]. To test this, the migration, angiogenesis, and immunomodulatory characteristics of hUCMSCs cultured in 2D and 3D were compared. Transwell assay results indicated that the number of migrating cells in the 3D-hUCMSC group was significantly higher than in the 2D-hUCMSC group (*P* < 0.05) (Fig. 4A), suggesting that 3D-cultured hUCMSCs exhibited stronger migratory capacity. Additionally, CM was collected from 2D (2D-CM) and 3D-cultured hUCMSCs (3D-CM) to assess their effect on the angiogenesis of HUVECs. The 3D-CM treatment on HUVECs resulted in a higher number of nodes and longer total tube branches (*P* < 0.05) (Fig. 4B), indicating a stronger tube formation and robust angiogenic response.

The immunomodulatory capability of the hUCMSCs was evaluated and compared between the 2D and 3D culture. qRT-PCR analysis revealed that the coculture with both 2D-hUCMSCs and 3D-hUCMSCs suppressed the gene expression of pro-inflammatory M1 markers (CD86, IL-1β, TNF-α, IL-6) (*P* < 0.05) and increased the gene expression of anti-inflammatory M2 markers (ARG-1, CD206) in macrophages (*P* < 0.05) (Fig. 4C). However, compared to the coculture with the 2D-hUCMSC group, the 3D-hUCMSC group showed better effects on suppressing the gene expression of pro-inflammatory M1 markers (TNF-α) (*P* < 0.05) and increased the gene expression of anti-inflammatory M2 markers (ARG-1, CD206, TGF-β1) (*P* < 0.05) (Fig. 4C). Meanwhile, flow cytometry results showed that coculturing with 3D-hUCMSCs up-regulated the population of CD206-positive (M2) macrophages and down-regulated CD86-positive (M1) macrophages compared to the 2D-hUCMSC group (*P* < 0.05) (Fig. 4D), indicating that 3D-hUCMSCs promote the transition of macrophages toward the M2 phenotype. In summary, our results indicate that 3D-cultured hUCMSCs have a more robust angiogenic and anti-inflammatory response compared to 2D-cultured hUCMSCs.

### Transcriptome comparison of 2D- and 3D-cultured hUCMSCs

**Fig. 5.**
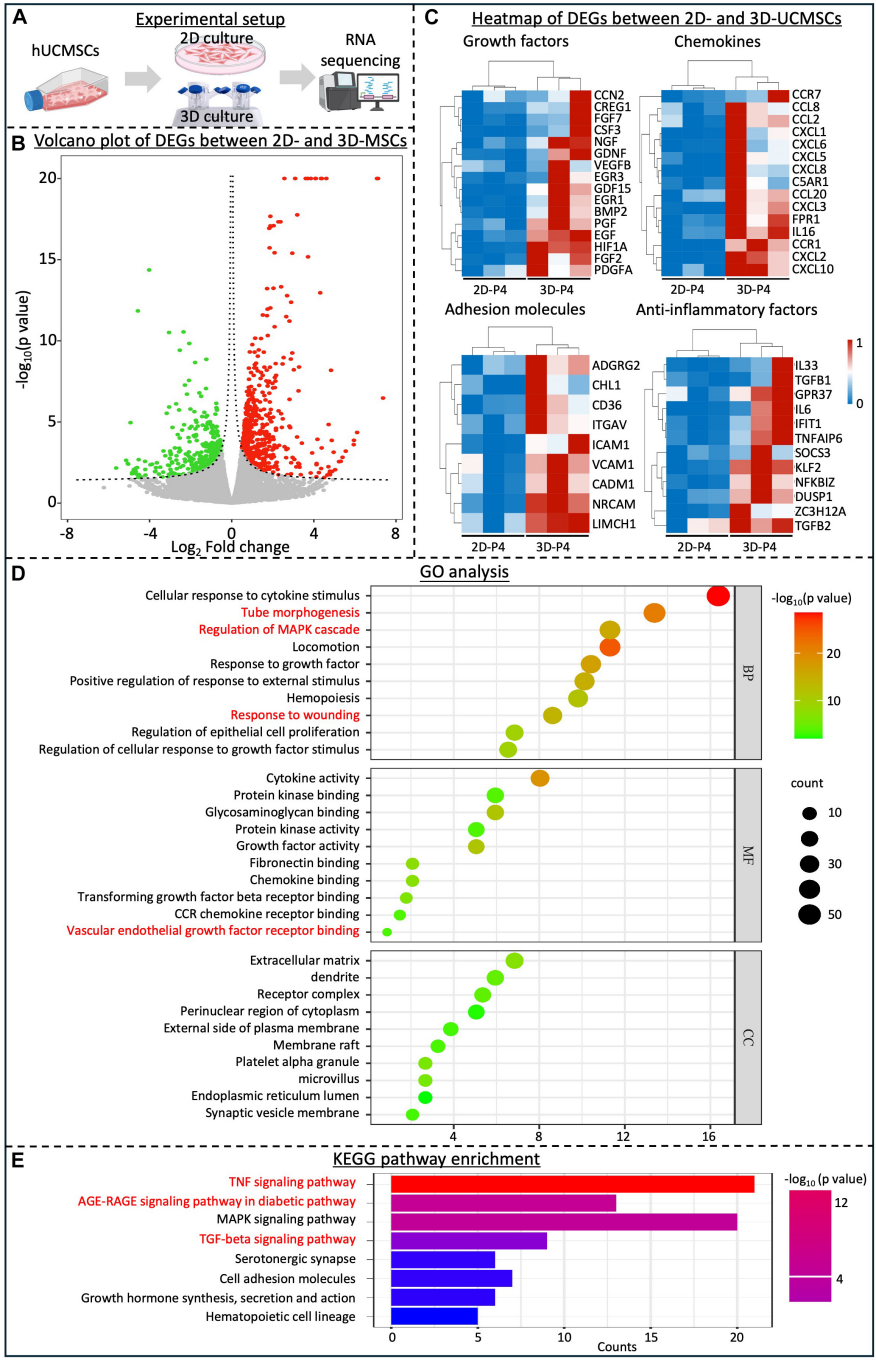
Differentially expressed gene profiles between 2D- and 3D-cultured hUCMSCs via RNA-seq. (A) Experimental setup of the RNA-seq. (B) Volcano plot of differentially expressed genes between 2D- and 3D-hUCMSCs. (C) Heatmap of differentially expressed growth factors, adhesion molecules, chemokines, and anti-inflammatory factor-related genes between 2D- and 3D-hUCMSCs. (D) GO clustering analysis of differentially expressed genes in 2D- and 3D-cultured hUCMSCs included 3 domains: biological process (BP), molecular function (MF), and cellular component (CC). (E) KEGG pathway enrichment of the signaling pathway enriched in the 3D-cultured hUCMSCs compared with 2D-cultured hUCMSCs (*n* = 3 independent assays).

To further compare the gene expression profiles and the activation of signaling pathways between 2D- and 3D-cultured hUCMSCs (at P4), RNA-seq was performed (Fig. 5A). The volcano plot and heatmap revealed differentially expressed genes between 2D- and 3D-UCMSCs (Fig. 5B and C). Compared to 2D-UCMSCs, 3D-UCMSCs exhibited significant up-regulation of several growth factors, adhesion molecules, chemokines, and anti-inflammatory factors (fold change ≥ 1 and *P* < 0.05) (Fig. 5C). Given the biological significance of these findings, we propose that 3D-hUCMSCs may exert their therapeutic effects through the up-regulation of angiogenic-related growth factors and immune-related genes. Target genes were subjected to KEGG pathway enrichment and GO clustering analysis. GO analysis revealed that most of the differential genes were positively related to angiogenesis, cell proliferation, protein kinase activity, and cytokine activity (Fig. 5D), while KEGG pathway analysis of differentially expressed genes indicated that differentially expressed gene (DEG) was involved in a variety of signaling pathways, such as advanced glycation end-products–receptor for advanced glycation end-products (AGE-RAGE) signaling pathway (diabetes related), TNF signaling pathway, and TGF-β signaling pathway (Fig. 5E). Our results suggest that 3D culture hUCMSCs showed significantly up-regulated expression levels of chemotaxis-, migration-, growth-, and anti-inflammatory-related genes and signaling pathways, which may improve their therapeutic efficacy.

**Fig. 6.**
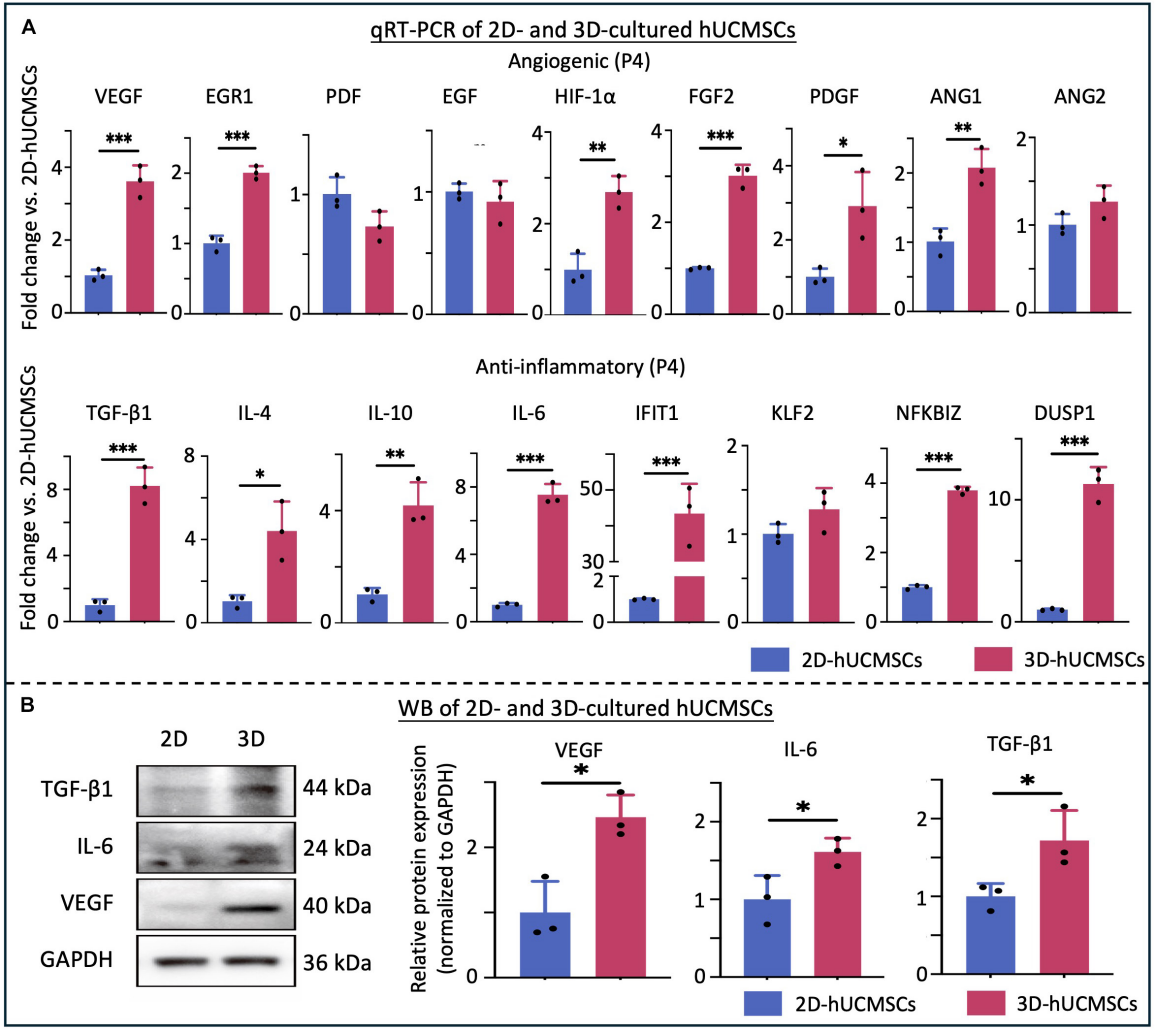
Assessment of angiogenic and inflammatory cytokines and growth factors in hUCMSCs cultured in 2D and 3D settings. (A) qRT-PCR analysis demonstrated significantly higher expression levels of angiogenesis genes, including VEGF, EGR1, HIF-1α, FGF2, PDGF, and ANG1, and anti-inflammatory cytokines, including TGF-β1, IL-4, IL-10, IL-6, IFIT1, NFKBIZ, and DUSP1, in 3D-cultured hUCMSCs compared to those cultured in 2D. (B) WB analysis showed increased protein levels of VEGF, TGF-β1, and IL-6 in 3D-hUCMSCs compared to 2D-hUCMSCs (mean ± SD; *n* = 3 independent assays, with each experiment comprising 3 technical replicates for every condition). *P < 0.05, **P < 0.01, ***P < 0.001.

To validate the findings from RNA-seq results and explore the expression of angiogenic and anti-inflammatory cytokines and growth factors in hUCMSCs cultured in both 2D and 3D settings, qRT-PCR and WB were performed. The qRT-PCR results demonstrated a significantly higher expression of angiogenic and anti-inflammatory genes, including VEGF, EGR1, HIF-1α, FGF2, PDGF, ANG1, TGF-β1, IL-6, IFIT1, NFKBIZ, DUSP1, IL-4, and IL-10, in 3D-cultured hUCMSCs compared to their 2D counterparts (Fig. 6A). Additionally, the WB analysis showed elevated protein levels of VEGF, TGF-β1, and IL-6 in 3D-cultured hUCMSCs compared to their 2D counterparts (*P* < 0.05) (Fig. 6B). These findings indicate that the 3D culture enhances the therapeutic potency of hUCMSCs, potentially by promoting angiogenic and anti-inflammatory effects.

### 3D-cultured hUCMSCs accelerated diabetic wound healing in mice

**Fig. 7.**
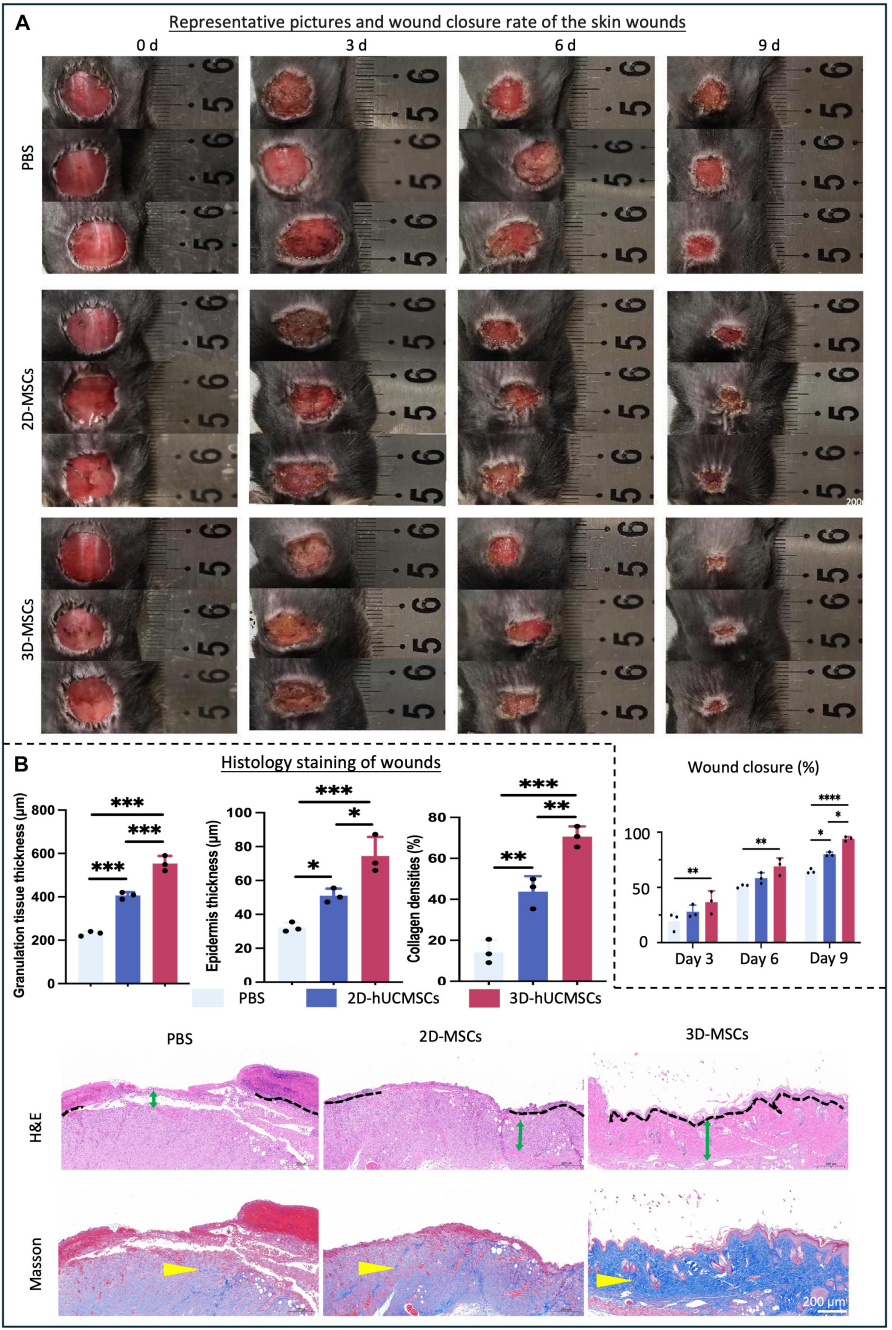
Assessment of wound healing in skin wounds treated with 2D- and 3D-cultured hUCMSCs in vivo. (A) Representative macroscopic images showed a faster wound closure rate at 3, 6, and 9 d post-treatment with 3D-hUCMSCs compared to 2D-hUCMSCs (mean ± SD; *n* = 3 donors; *P < 0.05, **P < 0.01, ****P < 0.0001). (B) Representative images of H&E and Masson’s trichrome staining on day 9. H&E staining and semiquantitative analysis showed that the epidermis in wound skin area was thicker, and the re-epithelization of the wound bed was more pronounced in the 3D-hUCMSC group compared to the 2D-hUCMSCs and PBS groups. The re-epithelialized areas are marked with black dashed lines, while the granulation tissue in the wound area is indicated by green double-sided arrows. Masson’s trichrome staining and semiquantitative analysis revealed that collagen fibers, highlighted by blue staining (marked by yellow arrows), were thicker and denser in the 3D-hUCMSC group compared to the 2D-hUCMSC group (mean ± SD; *n* = 3 donors; *P < 0.05, **P < 0.01, ***P < 0.001).

A full-thickness skin defect model in diabetic mice was established to investigate therapeutic effects of hUCMSCs on diabetic wound healing. One week after STZ treatment, mice received subcutaneous injections of 2D-hUCMSCs, 3D-hUCMSCs, or PBS around the defect area. The percentage of wound closure showed that the healing was slower in the PBS group compared to the treatment groups. The residual wound areas were significantly reduced in both the 3D-hUCMSC and 2D-hUCMSC groups at days 3, 6, and 9 compared to the PBS group (Fig. 7A). By day 9, the 3D-hUCMSC treatment group exhibited smaller wounds and thus better wound closure compared to the 2D-hUCMSC group (Fig. 7A). The therapeutic effects of different interventions of hUCMSCs were further evaluated with histological staining. Compared to the PBS-treated wound, the 3D-hUCMSC-treated wound was filled with more abundant newly formed granulation tissue (indicated by green double-sided arrows in Fig. 7B), along with an increased epidermal thickness and more pronounced re-epithelization of the wound bed, as evidenced by H&E staining and semiquantitative analysis (*P* < 0.05) (Fig. 7B). Additionally, Masson’s trichrome staining revealed that collagen fibers in the stem cell-treated groups were thicker and denser, characterized by a deep blue stain coloration indicative of enhanced collagen deposition (*P* < 0.05) (Fig. 7B). These results prove that the 3D-cultured hUCMSCs are more effective in promoting diabetic wound healing compared with 2D-cultured hUCMSCs.

### 3D-cultured hUCMSCs promoted angiogenesis and anti-inflammatory effect in diabetic wound healing

**Fig. 8.**
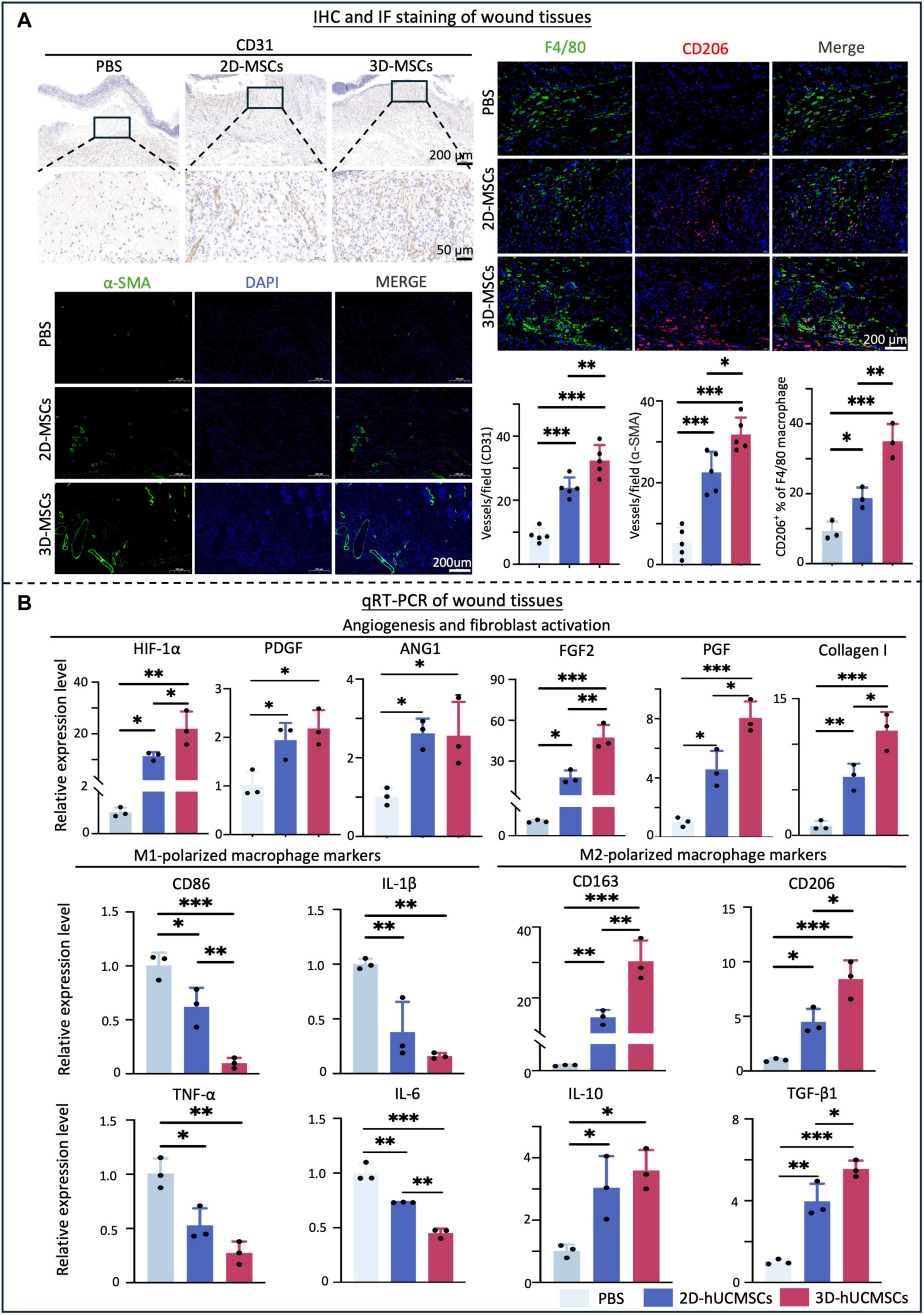
Assessment of angiogenesis and anti-inflammation effects of 2D- and 3D-cultured hUCMSCs on diabetic wound healing in vivo. (A) IHC staining demonstrated higher protein expression level of angiogenesis-related protein CD31 in the 3D-hUCMSC group. IF staining showed higher expression levels of angiogenesis-related protein α-SMA and M2-polarized macrophage-related protein CD206 in the 3D-hUCMSC group (mean ± SD; *n* = 3 donors; *P < 0.05, **P < 0.01, ***P < 0.001). (B) qRT-PCR analysis revealed higher expression levels of angiogenesis- and fibroblast activation-related genes HIF-1α, PDGF, ANG1, FGF2, PGF, and collagen I, and M2-polarized macrophage markers CD163, CD206, IL-10, and TGF-β1, and lower expression levels of M1-polarized macrophage markers CD86, IL-1β, TNF-α, and IL-6 in stem cell-treated groups compared to the PBS group, with GAPDH as the reference gene (mean ± SD; *n* = 3 donors; *P < 0.05, **P < 0.01, ***P < 0.001).

To evaluate the angiogenic and anti-inflammatory capabilities of 2D- and 3D-cultured hUCMSCs within diabetic wound healing, IHC, IF staining, and qRT-PCR were utilized. Neovascularization, detected by CD31-positive vascular endothelial cells, was observed in newly formed skin at the wound site on day 9. Quantitative analysis of the CD31-positive area in the wound tissue revealed that the number of blood vessels in the stem cell treatment groups was significantly higher than in the PBS control group (*P* < 0.05), with the 3D-hUCMSC group exhibiting a notably higher number of neonatal blood vessels compared to the 2D-hUCMSC group (*P* < 0.05) (Fig. 8A). Similarly, quantitative analysis of the α smooth muscle actin (α-SMA)-positive staining showed a significant increase in the stem cell group compared to the control group (*P* < 0.05) (Fig. 8A), with the 3D-hUCMSC group having higher expression level of α-SMA than the 2D-hUCMSC group, which further confirmed the increase of the neonatal blood vessels in the 3D-hUCMSC group. Additionally, on day 9, the RNA expression levels of angiogenesis-related factors (HIF-1α, ANG1, and PDGF) and fibroblast activation factors (FGF2, PGF, and collagen I) were significantly higher (*P* < 0.05) in the diabetic wounds treated with 2D and 3D-hUCMSCs than in the PBS control group. Notably, the 3D-hUCMSC group exhibited significantly elevated mRNA expression levels of HIF-1α, FGF2, PGF, and collagen I compared to the 2D-hUCMSC group (*P* < 0.05) (Fig. 8B).

The polarization of macrophages within the wound was assessed by IF staining. Quantitative analysis of M2-type macrophages in the wound tissue, through the expression of the M2 macrophage marker CD206, indicated a significantly higher expression in the stem cell-treated groups compared to the PBS group (*P* < 0.05), with the 3D-hUCMSC group showing more pronounced up-regulation of M2-type macrophages (*P* < 0.05) (Fig. 8A). The qRT-PCR results demonstrated that on day 9, the pro-inflammatory cytokines CD86, IL-1β, TNF-α, and IL-6 showed significantly lower expression levels in both hUCMSC-treated groups than in the control group. Notably, the expression levels of CD86 and IL-6 in the wound tissue of the 3D-hUCMSC group were significantly down-regulated compared to the 2D-hUCMSC group (*P* < 0.05) (Fig. 8B). In contrast, the anti-inflammatory cytokines CD163, CD206, IL-10, and TGF-β1 were significantly elevated in both hUCMSC-treated groups compared to the control group. Moreover, the expression level of CD163, CD206, and TGF-β1 in the 3D-hUCMSC group is significantly higher than that in the 2D-hUCMSC group (*P* < 0.05) (Fig. 8B). These results suggested that 3D-hUCMSCs may potentially modulate the inflammatory response by inducing M2-type macrophage polarization compared with 2D-cultured hUCMSCs.

## Discussion

MSCs possess the ability to differentiate into various cell types, migrate to their target niche (homing), and engage in paracrine signaling, rendering them a potent source for regenerative medicine [32]. However, generating an adequate quantity of high-quality MSCs ex vivo restricts the clinical translation of stem cell therapies. The DASEA bioreactor and microcarrier system can potentially address this challenge by providing an automatic and scalable technique for hUCMSC expansion. In this study, we comprehensively investigated the biological characteristics and potential therapeutic effects for diabetic wound repair of hUCMSCs produced in 2D and in 3D using the microcarrier-bioreactor system. Our results showed that compared to 2D cultures, 3D-cultured hUCMSCs significantly inhibited cell senescence while promoting cell migration, angiogenesis, and anti-inflammatory characteristics. These findings were further validated with RNA-seq and in vivo experiments, showing that 3D-cultured hUCMSCs exhibited enhanced therapeutic effects in diabetic wound healing compared to their 2D-cultured hUCMSCs with more pronounced angiogenic and anti-inflammatory effects.

The typical clinical dosing regimen for stem cell therapy ranges from approximately 0.5 × 10^6^ to 1 × 10^6^ cells per kilogram of body weight, with each patient requiring 4 to 8 administrations throughout the treatment course [33]. Unfortunately, the production yield of traditional 2D culture platforms is insufficient to meet these demands. This shortfall has driven the development of 3D culture platforms such as microcarriers and bioreactor combined culture systems. Studies by Nogueira et al. [34] and Rodrigues et al. [35] demonstrated that using the Vertical-Wheel bioreactors can achieve up to 10-fold higher expansion compared to conventional 2D culture systems. Meanwhile, microcarriers are also suggested as a promising tool to mitigate the limitations of monolayer cultures [36]. Microcarriers provides ample adhesion surfaces for MSCs in 3D suspension, allowing for cell expansion in a relatively small volume of culture medium [18,37,38]. Das et al. [39] suggested that microcarrier-based expansion significantly reduces medium usage while enabling high cell numbers in minimal volume. The single-step procedures of closed microcarriers bioreactor systems facilitate rapid cell expansion. The DASEA Regenbio bioreactor and microcarrier system exemplifies this approach to facilitate high-yield production of stem cells while offering cost-effective and time-efficient solutions. Additionally, the microcarriers are composed of AOF recombinant humanized collagen type I, which helps minimize the risk of introducing exogenous viruses. The digitalized, automated, and enclosed design of the bioreactors ensure uniform flow distribution, minimizes shear stress, and reduces cell damage, ultimately improving production efficiency and ensuring consistency in cell culture [22]. These advantages make this 3D microcarrier-bioreactor system particularly well-suited for industrial-scale MSC production, with high potential to enhance both the quality and therapeutic effectiveness of MSC-based treatments.

In addition to the significant clinical demand for cell numbers, maintaining cell quality during manufacturing poses a substantial challenge due to the high sensitivity of MSCs to their surrounding environment. A growing body of literature suggests that the properties of cell-seeding microcarriers, along with the dynamic flow in bioreactors that generate shear stress, and the frequency of collisions between cells and microcarriers, can significantly impact both cell growth and biopotency [12,18,40]. For example, studies have shown that physical properties like the shape and stiffness of microcarriers influence the yield and pluripotency of cultured stem cells [41]. It has been suggested that microcarriers used for the 3D expansion of UCMSCs should strike a balance between facilitating cell–cell contacts and enhancing cell–matrix interactions [42]. However, the role of biophysical signals and cell–matrix interactions in the context of 3D stem cell expansion remains not fully understood.

As a primary mechanistic evaluation, we compared gene expression between 2D- and 3D-cultured hUCMSCs in vitro. Findings from RNA-seq, qRT-PCR, and WB analysis demonstrated elevated expression levels of angiogenic and anti-inflammatory cytokines and growth factors. Moreover, the RNA-seq results revealed the activation of various tissue regeneration-related signaling pathways, such as TGF-β signaling pathway, in 3D-cultured hUCMSCs compared to 2D-cultured hUCMSCs. It has been reported that the activation of TGF-β signaling pathway is essential for effective and accelerated wound healing in skin tissues. TGF-β can cause ECM contraction, promote fibroblast differentiation into myofibroblasts, and increase collagen synthesis, thereby leading to wound healing [43]. Chemokines like C-X-C chemokine receptor type 4 (CXCR4), stromal cell-derived factor 1 (SDF-1), and C-C motif chemokine ligand 2 (CCL2) were increased in our 3D-cultured hUCMSCs and also play critical regulatory roles in (diabetic) wound healing, being closely associated with biological processes such as inflammation, cell proliferation and migration, angiogenesis, and matrix remodeling [44]. Ishida et al. [45] found that local application of CCL2 can promote neovascularization, collagen accumulation, and ultimately skin wound healing in diabetic mice. The increased expression of adhesion molecules such as vascular cell adhesion molecule-1 (VCAM-1) and intercellular adhesion molecule-1 (ICAM-1) was also observed in the 3D-hUCMSCs. These adhesion molecules play a crucial role in the homing process of stem cells. Specifically, when stem cells migrate to the blood vessels surrounding inflamed tissues, the ligands for these adhesion molecules expressed on the surface of stem cells can bind to integrins—adhesion factors released within the microenvironment. This interaction allows stem cells to enhance collateral vessel formation at the ischemic site, playing a key role in the pro-angiogenic functions of hUCMSCs [46]. Further exploration of these signal mechanisms is necessary to provide mechanistic rationale for the clinical application of 3D-cultured hUCMSCs.

In the rapidly advancing field of regenerative medicine, stem cell-based therapeutics hold great promise for skin wound regeneration [47]. To meet the increasing clinical demand for stem cell production, various 3D cell culture systems have been developed, including conventional stirred tank bioreactors. Additionally, widely employed platforms such as fixed and fluidized-bed systems (e.g., packed-bed and fluidized-bed bioreactors) and disposable bioreactors [e.g., CellCube (Corning), Cell Factory (Nunclon), Regenbio (REGEN GEEK), and the Wave bioreactor (WaveBiotech)] are becoming standard in the industry. Innovative designs such as airlift and filtration-based designs (e.g., spin, hollow-fiber, acoustic, and crossflow filters) also show promise for enhancing cell culture efficiency and scalability [36,48]. To date, over 1,000 clinical trials involving MSCs have been registered on clinicaltrials.gov. However, fewer than 10 of these studies used bioreactor-cultured MSCs and most of them are in phase I. Despite advancements in 3D-cultured hUCMSCs, the clinical translation of these therapies faces several challenges. A critical issue is the lack of standardized procedures for industrial cell culture, which can lead to variability in cell quality. This is particularly important in 3D stem cell manufacturing that involves more complex procedures and conditions than traditional 2D cultures, such as the heterogeneity caused by discontinuous growth surfaces and uneven shear stress distribution within bioreactors [49]. Establishing standardized procedures for stem cell culture and implementing rigorous QA and quality control measures at various checkpoints are essential for ensuring consistent quality and quantity for clinical application of the hUCMSCs. The scientific and clinical significance of our study lies in the comprehensive characterization of the safety, stability, and efficacy of hUCMSCs expanded using a 3D microcarrier-bioreactor system for diabetic wound healing. Our findings indicate that 3D expansion of hUCMSCs offers distinct advantages over traditional 2D culture, presenting a reliable and translatable approach for clinical cell therapy with enhanced therapeutic potential. By addressing the challenges of standardization and quality control, we can facilitate the successful implementation of hUCMSC therapies, ultimately improving patient outcomes in regenerative medicine.

## Data Availability

All data that support the findings of this study are included within the article (and any supplementary files).
